# Inhibitory Neuron and Hippocampal Circuit Dysfunction in an Aged Mouse Model of Alzheimer's Disease

**DOI:** 10.1371/journal.pone.0064318

**Published:** 2013-05-17

**Authors:** Anupam Hazra, Feng Gu, Ahmad Aulakh, Casey Berridge, Jason L. Eriksen, Jokūbas Žiburkus

**Affiliations:** 1 Department of Biology and Biochemistry, University of Houston, Houston, Texas, United States of America; 2 Department of Pharmacological and Pharmaceutical Sciences, University of Houston, Houston, Texas, United States of America; University of South Florida Alzheimer's Institute, United States of America

## Abstract

In Alzheimer's disease (AD), a decline in explicit memory is one of the earliest signs of disease and is associated with hippocampal dysfunction. Amyloid protein exerts a disruptive impact on neuronal function, but the specific effects on hippocampal network activity are not well known. In this study, fast voltage-sensitive dye imaging and extracellular and whole-cell electrophysiology were used on entorhinal cortical-hippocampal slice preparations to characterize hippocampal network activity in 12–16 month old female APPswe/PSEN1DeltaE9 (APdE9 mice) mice. Aged APdE9 mice exhibited profound disruptions in dentate gyrus circuit activation. High frequency stimulation of the perforant pathway in the dentate gyrus (DG) area of APdE9 mouse tissue evoked abnormally large field potential responses corresponding to the wider neural activation maps. Whole-cell patch clamp recordings of the identified inhibitory interneurons in the molecular layer of DG revealed that they fail to reliably fire action potentials. Taken together, abnormal DG excitability and an inhibitory neuron failure to generate action potentials are suggested to be important contributors to the underlying cellular mechanisms of early-stage Alzheimer's disease pathophysiology.

## Introduction

Alzheimer's disease (AD) is the most common form of dementia in patients over the age of 65 that manifests as a progressive degenerative disorder in the central nervous system. AD is predominantly associated with a progressive decline in cognitive abilities that first manifests as word finding difficulties and impairments in short-term memory [1]. In addition to gross cortical atrophy, the pathological hallmarks used to definitively identify Alzheimer's disease include the presence of insoluble extracellular amyloid protein and intracellular neurofibrillary tangles [2]–[6]. While the specific pathologies that lead to cognitive disruption are unknown, current theories favor the idea that amyloid protein forms into toxic amyloid oligomers and fibrillar aggregates that promote the development of tau hyperphosphorylation, ultimately resulting in neuronal dysfunction and death [7]–[9].

Although the exact processes that contribute to initial development of Alzheimer's disease are not entirely known, a multitude of studies over the past two decades have suggested that the accumulation of the amyloid beta (Aβ) protein is a critical contributor to the development of early cognitive dysfunctions, such as memory loss, seen in the earliest stages of Alzheimer's disease [10]. Although most transgenic mouse models of amyloid pathology show no signs of cell death, the majority of these lines demonstrate numerous behavioral abnormalities and many display cognitive dysfunctions that follow the accumulation of amyloid protein [11]. Notably, most transgenic lines show progressive impairments in hippocampal-based spatial memory; these impairments have been associated with Aβ associated synaptic dysfunction [9], [12]–[14]. Recently, a variety of studies have begun to describe effects of amyloid, not only at the synaptic level, but within a broader network activity characterization. Studies of AD patients indicate that the development of the disease is positively correlated with epileptic activity [15]; similarly, different lines of amyloid-expressing transgenic mice also show spontaneous epileptic seizures. Studies of several different lines have demonstrated spontaneous epileptiform activity which has been attributed to increased network hyperexcitability, particularly in cortical regions [16]–[21], suggestive of broad network changes. To date, relatively little is known about changes in neural activity propagation dynamics in the hippocampal memory circuits, either during aging or in AD [21]–[23]. The limbic networks primarily mediate memory encoding, whereas the cortico-hippocampal circuitry is thought to serve as an anatomical entry gate for forming memory traces. Within the rodent hippocampus, perforant pathway projections into the dentate gyrus carry the most prominent cortical inputs responsible for pattern separation [24] and spatial information and memory encoding [25]. The hippocampus encodes and consolidates these memory traces through oscillations, created by finely tuned action potential firing of both excitatory and inhibitory cells [26]–[28]. However if either inhibitory or excitatory cells do not function properly, this may affect neural oscillations, information processing and memory encoding.

Recent advances in imaging techniques have allowed for the identification of large-scale functional interactions between heterogeneous brain regions. For example, fast voltage-sensitive dye imaging (VSDI) allows for the measurement of the membrane potential of neurons across wide spatial areas with fast temporal and spatial resolutions [29]–[31]. VSDI signals are linearly calibrated to changes in neuronal membrane potential and can be performed with extracellular or single cell recordings [29], [32]–[35]. This offers unprecedented opportunities to study and identify disruptions of neural activity in key anatomical circuits associated with normal aging and the onset and progression of AD [36]–[38]. While nearly all published studies have used tissue from young animals for VSDI imaging, in this study using concurrent electrophysiology and VSDI in aged brain tissue, we show that Aβ expression results in broadly disruptive changes in hippocampal circuit and cell excitability, resulting in impaired short-term synaptic plasticity (STP) in the dentate gyrus. These findings suggest that the disruption in the inhibitory cell and network activity is likely to be an important contributor associated with the decline in spatial memory observed in APdE9 and similar hAPP mouse lines and, more broadly, these findings also suggest that amyloid protein may directly contribute to the decline in short-term memory associated with Alzheimer's disease.

## Materials and Methods

### Animals

**Figure 1:**
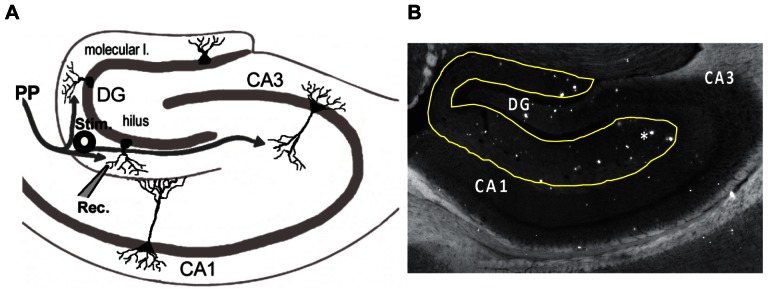
Experimental set-up and a typical plaque distribution in the hippocampus of aged APdE9 Mice. (A) Cartoon of the hippocampal circuit and the major connecting pathways. DG – dentate gyrus, EC – entorhinal cortex, CA – cornus ammoni, PP and the grey arrows – perforant pathway projections from the entorhinal cortex. Stimulating electrode (Stim.) was placed on the medial PP and recordings were performed in the apical dendritic field of the granule cells. (B). Photomicrograph of the hippocampus of APdE9 mouse. At 12–16 months, brains of the model mice contains a substantial Aβ plaque burden (*) in the hippocampal areas. In the hippocampus, plaque deposit is high in the molecular layer of the DG (approximate boundaries are outlined in yellow), the site of perforant pathway projections. Scale bar −300 µm.

Studies were performed on 12–16 month old female mice with mutant human APPswe and PSEN1DeltaE9 genes (APdE9) and age-matched non-transgenic siblings (NTG), Fig. 1B [39], [40]. These animals are significantly impaired in spatial memory performance by 12 months in the absence of cell death [39], [40].

### Ethics Statement

This study was carried out in strict accordance with the recommendations in the Guide for the Care and Use of Laboratory Animals of the National Institutes of Health. The protocol (Permit Number: 08–035) was approved by the University of Houston's International Animal Care and Use Committee.

### Entorhinal cortical-hippocampal slice preparation

The mice were anaesthetized with isoflurane and decapitated, and the brains were rapidly excised and placed in oxygenated (95% O_2_-5% CO_2_), ice-cold dissection buffer solution containing (in mM) 212.7 sucrose, 2.5 KCl, 1.25 NaH_2_PO_4_, 3 MgSO_4_, 10 MgCl_2_, 0.5 CaCl_2_, 26 NaHCO_3_, and 10 dextrose. Hippocampal-entorhinal cortical slices (350 µm) were prepared using a Vibratome (Technical Products International) and preincubated for 0.5 h in normal artificial cerebrospinal fluid (ACSF; pH 7.3, 30°C) containing (in mM): 130 NaCl, 1.2 MgSO_4_, 3.5 KCl, 1.2 CaCl_2_, 10 glucose, 2.5 NaH_2_PO_4_, and 24 NaHCO_3_ aerated with 95%O_2_-5%CO_2_.

### Optical imaging and electrical recordings

Following the 30 minute pre-incubation of the slices, 60 µl of voltage-sensitive dye di-4ANNEPS (Sigma-Aldrich) stock solution were applied over the slice surfaces with a final dye concentration of 0.03 mg/ml [35]. Slices were incubated in ACSF with the dye for an hour before recording began [41], [42].

### Extracellular recordings and imaging

We used a combination of *in vitro* electrophysiology (extracellular field potential recording and whole-cell patch clamp recordings) and fast voltage-sensitive dye imaging (VSDI). After the incubation, slices were placed in a submersion chamber (Warner Instruments) under the upright wide-field epifluorescence microscope (BX51WI; Olympus) equipped with a complementary fast CCD camera for VSD imaging (128×198 pixels, MiCam 02, SciMedia, USA) and an infrared-differential interference contrast (IR-DIC) camera (Dage MTI) for visualized whole cell recordings. Slices were continuously perfused (2 ml/min, at 30°C) with oxygenated ACSF. Extracellular recordings were performed using borosilicate glass electrodes filled with 0.9% NaCl (1–2 MΏ) simultaneously with VSDI. A concentric bipolar stimulating electrode (FHC, 200 µm diameter) was placed on the medial perforant pathway fibers entering from the entorhinal cortex into the dentate gyrus (DG) of the hippocampus and electrical field excitatory post-synaptic potentials (fEPSP) were recorded in the apical dendrites of the granule cells located in the molecular layer of the DG (Figure 1A). Synaptic responses were evoked at a 40 Hz frequency, which in the hippocampus is associated with the gamma rhythm, often activated during rodent exploration of a novel environment [27]. Half maximal amplitudes of the evoked response were used to study fEPSP responses during 40 Hz stimulation. 40 Hz stimulation consisted of 10 pulses at 40 Hz repeated every 30 seconds.

Extracellular recordings were performed concurrently with the VSDI. Stained slices were illuminated with electronic shutter controlled 150 W halogen light source (Moritex Corp.) and passed through a filter cube (excitation λ 530±10 nm, dichroic λ 565 nm and absorption λ>590 nm, U-MWIG2, Olympus). Evoked optical signals were acquired at 250 Hz optical sampling rate. To increase optical and electrical signal to noise ratio for the stimulation experiments, averages of ten trials were used when quantifying fEPSP amplitudes and optical signal characteristics in each slice. To measure effects of this ten pulse (Pulse 1-Pulse 10) 40 Hz stimulation on the short-term synaptic plasticity (STP), we compared the response characteristics produced within the stimulation train. To calculate the spatial extent during the synaptic stimulation, fast optical signals were characterized by the spatial extent of the evoked neural activity using fractional change in fluorescence signal (ΔF/F_max_) and calculated by comparing the change in the intensity of fluorescence (ΔF) in each pixel relative to the maximum intensity of background fluorescence (F_max_) [35]. Activation maps of the average files in each slice were generated using the threshold based function in Brain Vision software. Identical gain and threshold parameters were used when comparing optical data in all experimental conditions. Extent of spatial activation was measured as the number of active pixels that reached 50% or more of its normalized average maximal activation value.

### Whole-cell recordings in the aged dentate gyrus interneurons

To study individual inhibitory neuron activity, we performed whole-cell recordings in the inhibitory cells of the dentate gyrus molecular layer. Inhibitory neurons were visualized and initially identified based on the location and shape of their somatas using infrared optics. For the whole cell current-clamp recordings, micropipettes (4–7 MΩ) contained: 116 mM K-gluconate, 6 mM KCl, 0.5 mM EGTA, 20 mM HEPES, 10 mM phosphocreatine, 0.3 mM NaGTP, 2 mM NaCl, 4 mM MgATP, and 0.3% neurobiotin (pH 7.25, 295 milli-osmolar). All electrical recordings were performed using MCC 700 amplifiers (Axon Instruments). Whole-cell data were low-pass filtered at 4 kHz and digitized at 10 kHz (Digidata; pCLAMP; Molecular Devices). Passive and active neuronal membrane properties were studied using incremental hyperpolarizing and depolarizing current injections. To elicit spiking activity, depolarizing square wave current pulses incremented by 20 pA were injected into the somas for 500 ms, followed by 10 ms return to the baseline holding membrane potential (−70 mV), and then a gradual ramp depolarization (10–40 mV/100 msec). This ramp protocol allowed us to test whether brief hyperpolarization of the cell membrane was efficient at restoring reliable action potential firing.

### Neuroanatomy

Amyloid deposits were identified *post hoc* using 5 minute staining in 1% thioflavin-S followed by 5 minute differentiation in 70% ethanol. For immunofluorescence studies, 300 µm tissue sections used in imaging studies were fixed overnight in 4% PFA. After sections were washed with Tris-buffered saline (TBS) containing 0.5% Triton X-100 solution (TBST), tissue was treated with a Cy5-labeled streptavidin (Vector Laboratories, Burlingame, CA) solution for 1 hour in order to label cells filled with neurobiotin during electrophysiological studies. Sections were treated with an avidin/biotin blocking kit (Vector Laboratories, Burlingame, CA) and then incubated in TBS, 5% bovine serum albumin (BSA), 0.5% Triton X-100 for 1 hour as a blocking step. Sections were double labeled with GAD65 (1∶1000; AB5082, Millipore) or GAD67 (1∶1000; MAB5406, Millipore) and antibodies overnight at 4°C. Sections were incubated with biotinylated goat anti-rabbit antibody (Vector Laboratories, Burlingame, CA) for 60 min, washed with TBST, and then incubated with Texas Red Avidin DCS (Vector Laboratories, Burlingame, CA) for 20 minutes. Sections were washed with TBST, re-blocked using the avidin/biotin blocking kit, subjected to second round of serum blocking, and then incubated with a second round of antibodies, followed by washing and incubation with Fluorescein Avidin (Vector Laboratories, Burlingame, CA) for 10 min. Sections were mounted in Vectashield media (Vector Laboratories), and viewed under a confocal microscope (Olympus IX61 DSU). Images were processed with Neurolucida (Microbrightfield) in order to confirm localization and reconstruct the morphology of stained cells; dendrites and axons were traced using Neurolucida's neuron-tracing software.

### Data and statistical analysis

Optical and electrical data were analyzed using Brain Vision and pClamp software. Same threshold and gain settings were used for all of the optical data representation. Results on network activation are based on the measurements of the fEPSPs and reported as grouped averages with standard error of the mean. Statistical significance was tested using unpaired t-test analysis. A p value of <0.05 was regarded as statistically significant.

## Results

### Hippocampal Pathology

APdE9 mice develop significant impairments in spatial memory by 12 months of age [39], [40]. In this study, slices used for the study at the ages 12–16 months had a significant plaque burden in the hippocampus of the APdE9 transgenic animals. Amyloid deposits were visualized after imaging using standard anatomical staining technique with Thioflavin-S, and also appeared visible using the infrared optics. Major plaque deposit could be seen throughout the key hippocampal circuits, including surrounding the major neocortical input pathway, the perforant pathway of the dentate gyrus (Fig. 1). The majority of the plaques could be observed outside of the granule and pyramidal cells layers, concentrated in the molecular and hilus of dentate gyrus, and the radiatum and oriens of CA. In our studies, we stimulated the medial perforant pathway (Fig. 1A) which innervates the DG and the lacunosum-moleculare and upper radiatum layers in the CA1. Molecular and hilar layers of DG are known to be comprised of up to 90% of the inhibitory interneurons [43].

### Impaired Short-Term Synaptic Plasticity in Aged APdE9 Mice

**Figure 2:**
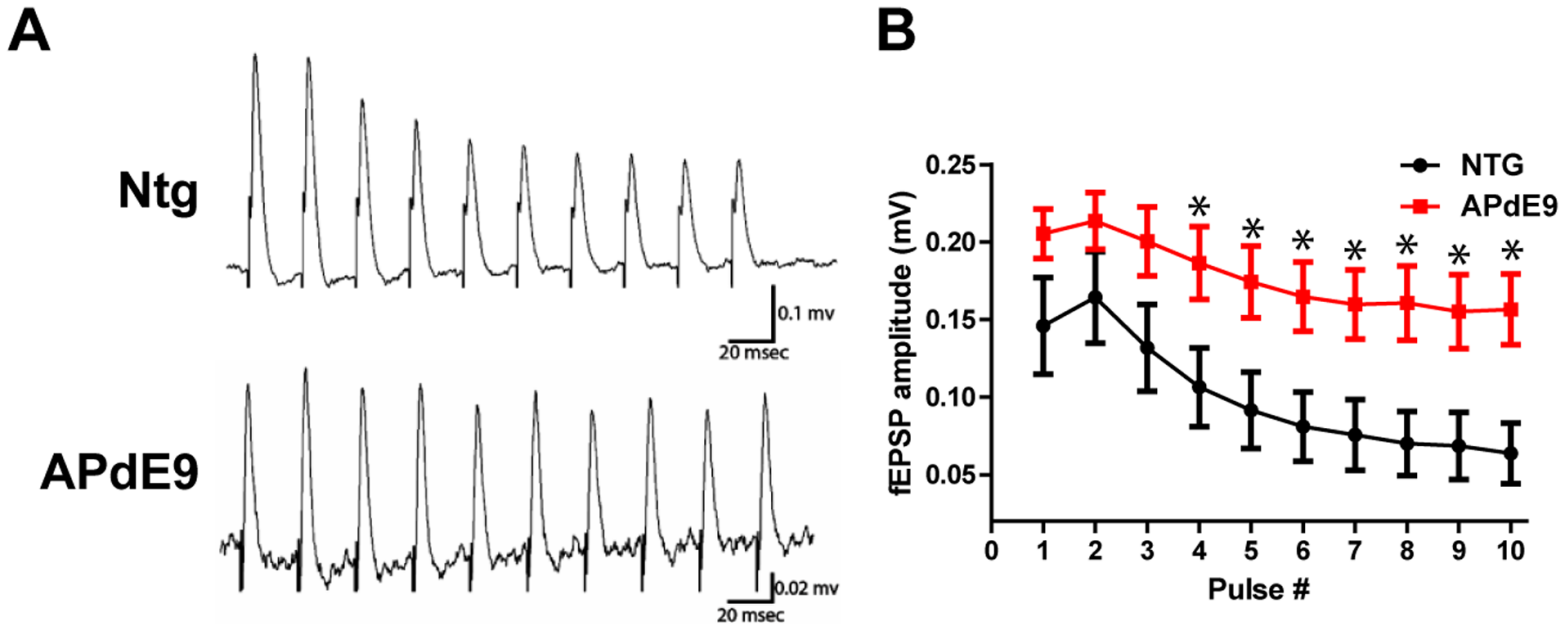
Impaired STP in the DG of APdE9 mice. (A). Representative field potential responses recorded extracellularly in NTG mouse tissue. 40 Hz 10 pulse stimulation evoked depressive (decreasing amplitude) responses in the NTG animals. APdE9 mice showed sustained field potential responses with little decrease in the amplitude throughout the stimulation train. (B) Average and S.E.M. for APdE9and NTG fEPSP amplitudes during the 40 Hz stimulation. Note significant differences for the fEPSP amplitudes produced by pulse stimulations 4–10 (pulse to pulse comparison, unpaired t-test, p<0.05).

To determine if there were any changes in the synaptic short-term plasticity, we measured amplitudes of the evoked field EPSPs during the short 40 Hz frequency stimulation trains of the perforant pathway. Half maximal stimulation amplitudes yielded larger amplitude responses in the APdE9 tissue. These responses were also sustained during the stimulation trains (Fig. 2). The difference in the amount of field potential activity is especially evident during the second half of the stimulation train (Pulses 4–10). We found that although both the early and the late synaptic responses were depressed (below 1), responses from the wild-type aged tissue was significantly more depressed than in the APdE9 perforant pathway circuit.

### Hippocampal Circuit Hyperexcitability in the Dentate Gyrus of APdE9 mice

**Figure 3:**
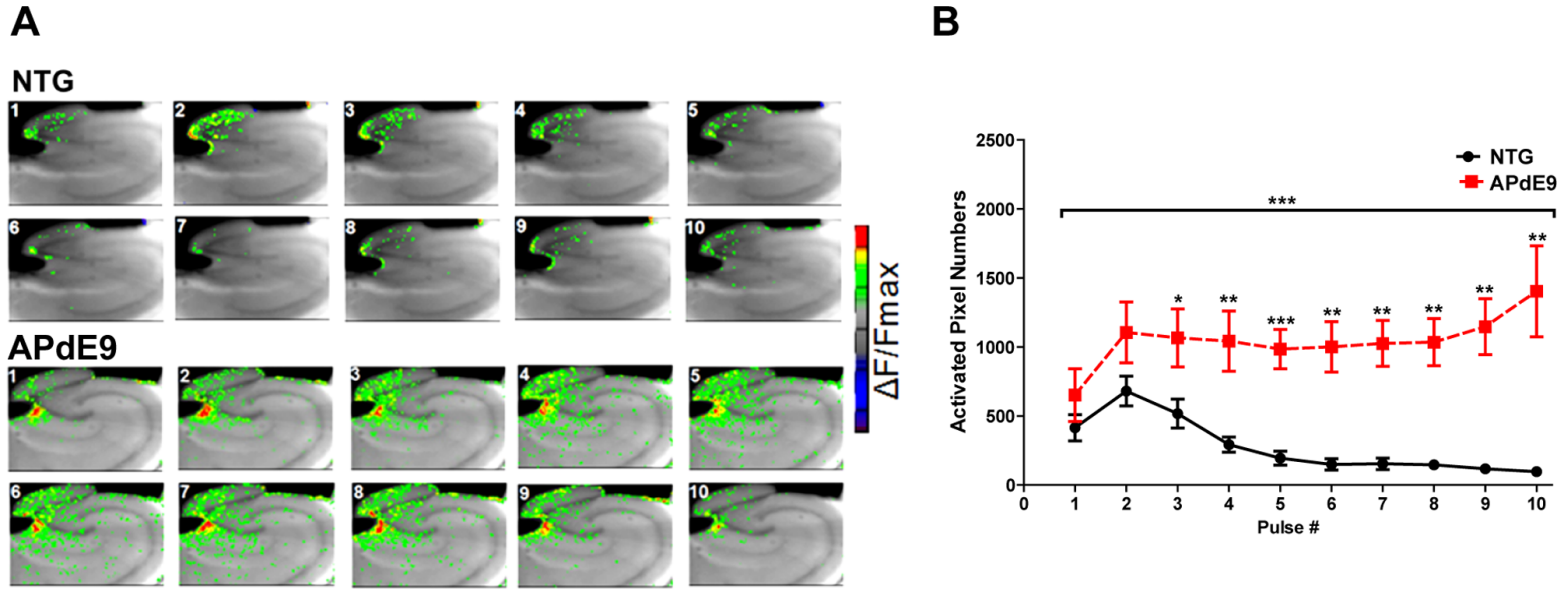
Imaging dentate gyrus hyperexcitability during 40 Hz stimulation. (A) VSDI during 40 HZ stimulation in APdE9 (top panel) and NTG tissues (bottom panel) (acquired at 250 Hz). Photomicrographs depict transverse slices of the hippocampus overlaid with the normalized average (10 trials) VSD signals. Thick black line shows the stimulating electrode (200 µm tip) and the site of perforant pathway stimulation in the DG. Frames 1–10 correspond to the time of the peak of 10 fEPSP responses. 40 Hz train stimulation in the NTG tissue evoked a typically small and concise neuronal activity map. In APdE9 mice, equivalent amplitude stimulation evoked wide and non-specific signal spread. Scale bar  = 250 µm. (C) Average optical signal quantification based on number of activated pixels above 50% threshold level (Methods) showed that PP stimulation in APdE9 mice evoked significantly larger neural activity maps. (N = 7 APdE9, 7 NTG; p<0.0001, unpaired t-test).

Concurrently with extracellular electrophysiology (Fig. 2), we performed fast VSDI experiments (Fig. 3) to examine functional neural circuit characteristics. To our knowledge, this is the first visualization of the neural activity profiles in the hippocampus of aged AD mice using the VSDI technique. During 40 Hz stimulation, APdE9 tissue showed significantly different spatial hippocampal network activation profiles when compared with the age-matched non-transgenic (NTG) animals. APdE9 activation at the same stimulation amplitudes was substantially larger as compared to the activity in the NTG hippocampus. In NTG tissue, electrical stimulation produced neural activity that was relatively confined to the perforant pathway activation in the dentate gyrus. The 40 Hz stimulation in the APdE9 mice activated significantly larger areas with significant activation of the CA3 area in addition to the DG. Consistent with changes in electrical responses (Fig. 2 and 3A), optical responses were increased in size and showed a significant difference in the area of activation starting from the third pulse in the ten pulse stimulation train. These findings indicate that DG gating of the neural input is impaired in the APdE9 phenotype, leading not only to sustained activation of the DG, but also anatomically non-specific activation of the adjacent hippocampal pathways.

### Dysfunctions in the Inhibitory Cells of the Dentate Gyrus

Increases in the fEPSP responses as well as the imaging results appeared to be remarkably similar to the altered DG function, described as a “broken DG gate,” reported the chronic models of epilepsy [30], [31]. In models of chronic epilepsy, altered network activity is often attributed to impaired inhibitory neuron function [44], [45]. Consequently, we attempted to determine if breakdown of the DG gate in the Aβ model of AD was similarly caused by impaired activity of inhibitory interneurons.

**Figure 4:**
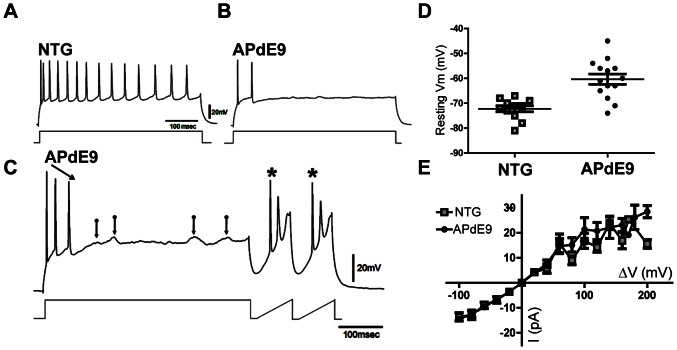
Inhibitory DG Cells Fail to ‘Spike’. Examples of electrical recordings from NTG and APDE9 inhibitory interneurons. (A) NTG interneuron membrane responses following positive current injection. (B) When depolarized incrementally, APdE9 interneurons showed dramatic failures at producing continuous trains of action potentials. (C) In some instances the spikes could be recovered (*) with hyper-polarization following the depolarizing current injections, indicating a possible channelopathy. (D) Interneuron resting membrane potentials (V_m_) in NTG (open squares) and APdE9 (filled circles) tissues were significantly different (NTG: V_m_ = −72.73±1.176 N = 11; APdE9: V_m_ = −60.36±2.082 N = 14, p<0.0001, unpaired t-test). (E) Current vs. voltage plots, depicting passive membrane properties of the input resistance (R_in_). Positive and negative square wave pulses were injected while the cells were held at −70 mV in a current clamp mode. Electrode resistance was bridge balanced and despite the difference in the V_m_, R_ins_ in NTG and APdE9 interneurons were not significantly different (p = 0.558, Welch's t-test, df = 30).

Normal brain activity and learning is an exquisite balance of excitatory and the inhibitory cell activity. Many of the important neural rhythms, including theta and gamma (40 Hz), require precise action potential firing from discrete subpopulations of the inhibitory neurons. The molecular layer of the DG almost exclusively contains inhibitory interneurons [43]. These cells are responsible for feed-forward inhibition; and the outputs of these interneurons project into the perforant pathway fibers or into the perisomatic regions of the granule cells, thereby exerting a powerful control on their excitability. Using whole-cell patch clamp in vitro (Fig. 4), we found that 65% (9/14) of the patched cells in aged APdE9 failed to reliably fire actions potentials, whereas only 22% (3/9) of the cells showed action potential failures in the age-matched NTG animals (Fig. 4). This finding suggests that a decline in inhibitory neuron function is a process of normal aging that is exacerbated in the presence of Aβ accumulation, and the hyperexcitability observed in APdE9 mice is strongly associated with a decrease in inhibitory neuron activity.

In addition to the failure of the action potential firing, we also found that the molecular layer cells in the APdE9 mice had significantly depolarized resting membrane potentials (Fig. 4D; n = 14 APdE9, n = 11 NTG). This would suggest that the cell membranes are potentially leaky, leading to a loss in ionic charge separation between the intra and extracellular sides of the cell membrane. To test this, we measured input resistances (R_in_) in response to the hyperpolarizing and depolarizing square wave current injections. The R_in_ (I–V) curves did not show significant differences, suggesting that depolarized resting membrane potential in the APdE9 interneurons is likely not due to the membrane leakiness.

**Figure 5:**
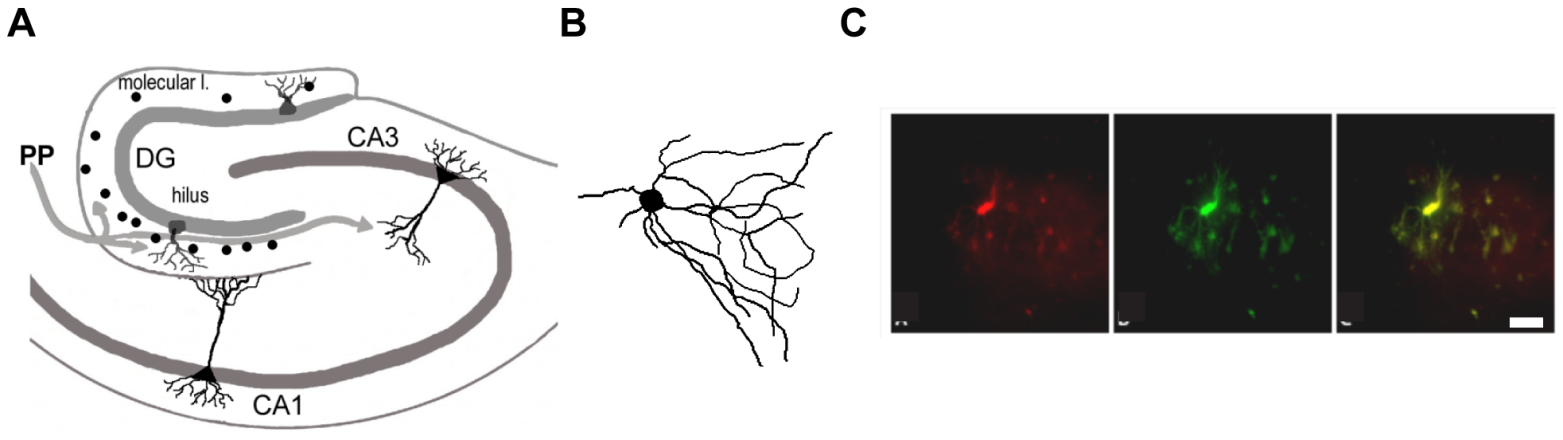
Identification of the inhibitory interneurons. (A) Cartoon of the hippocampus. Black dots in the molecular layer of the DG indicate the locations of recovered cells stained following the electrophysiological recordings. (B) An example of recorded interneuron which was reconstructed post-hoc using neurobiotin immunohistochemistry. (C) An example of the recorded identified interneuron that stained positively for neurobiotin (left, red) and GAD (middle, green) stains (interneuron marker). Right: Neurobiotin and GAD overlapped showing co-localization (right, yellow). Scale bar  = 10 µm.

Post-hoc, we recovered the majority of the recorded cells and confirmed that they were inhibitory interneurons by double staining the recorded cells for the neurobiotin and GAD antibodies. Approximate locations, reconstructed anatomy, and cross-staining photomicrographs of the recorded cells are shown in Figure 5.

## Discussion

Early stages of AD present as a temporary loss of memory or inability to form new memories. Growing evidence also suggests that early pathology at the synapses and synaptic terminals may precede the gross anatomical changes such as substantial cell loss and selective loss of key neural circuits [46]. Consequently, it is critical to understand the molecular and cellular mechanisms that lead to the breakdown of the normal neural circuit function, initiating the process of dementia. The results described here provide evidence that Aβ expression results in severe neural activity dysfunctions in the limbic-hippocampal circuits, including impaired short-term plasticity and neural circuit hyperexcitability. We further demonstrate that this hyperexcitability is associated with the inability of the inhibitory interneurons to reliably generate action potentials, resulting in impaired DG function that is analogous to that observed in epilepsy [47]; the loss of inhibitory interneuron function is likely to be an important contributor to the seizures reported in the APdE9 line [16].

### Altered synaptic plasticity in Alzheimer's disease

Although it is well accepted that dynamic and chronic changes in the cellular substrates can alter synaptic plasticity, changes in different forms of synaptic plasticity during the process of dementia are not well understood [23], [48]. Majority of the studies in AD models have concentrated on the long-term form of synaptic plasticity induced by prolonged high frequency stimulation. Only a handful of studies have investigated changes in short-term plasticity in the AD models induced using ‘rate-code’ stimulation paradigms [49]–[51] but little is known how short term and spike-timing dependent plasticity is affected in the AD models. Previous reports from CA3 and CA1 areas of the hippocampus show that either the acute application of Aβ protein or the progression of Aβ pathology significantly affects excitatory neurotransmitter release probability, causing synaptic depression or reducing synaptic facilitation. The predominant findings in the synaptic plasticity studies are that Aβ protein reduces long-term potentiation and/or causes synaptic depression [9]. The relationship between the depressing synapses and hyperexcitability observed in APP models has been difficult to reconcile [48]. Our results show that in the perforant pathway there is a substantial increase in the synaptic facilitation, synonymous with the increased network activation. It remains to be determined if the differences observed in facilitation or depression could be in part dependent on the synaptic circuit studied, the model used, or the age at which the recordings are performed. Fast functional imaging and optical control tools may become critical in dissecting out the functional breakdown in multiple circuits and distinct cell subtypes, and correlating these findings with the molecular and behavioral level alterations.

### Power of imaging to unravel synaptic AD circuit dysfunctions

Using VSDI (Fig. 1C) and extracellular electrophysiology (Fig. 2), we discovered that accumulation of Aβ along the perforant pathway (PP) profoundly disrupts hippocampal activity, showing increased excitatory synaptic responses and spatiotemporal hyperexcitability of the DG perforant pathway circuit. Increased synaptic excitation is in agreement with previous reports of hyperexcitability in the Aβ model and could be due to the molecular changes [13], increased number of the excitatory synapses or the hyperactive cells located in the proximity of the Aβ plaques [22]. Fast VSDI allowed us to visualize the neural circuit activity in the aged APdE9 and NTG mice. Our results show that Aβ pathology causes runaway excitation which permeates into the hippocampal circuits adjacent to the dentate gyrus, described as the ‘breakdown of the dentate gate’ [52]. Extraordinarily, a similar breakdown of the DG gate has been previously observed in the chronic forms of epilepsy [30], [31], [53]. Consequently, it is likely that the abnormal spatiotemporal activation of the hippocampal networks is not only contributing to the hyperexcitability, but also impairing normal neuronal activity of processing and encoding information, events that contribute to impaired synaptic plasticity. Given that these events are necessary for the proper function of learning and memory, it is likely that these alterations contribute to the impaired spatial memory observed in the APdE9 and other hAPP mouse models.

### Hyperexcitability in dementia

The hyperexcitability seen in the animal models of AD closely parallel the human condition. Longitudinal studies of aging populations have established that the risk of seizure becomes dramatically elevated in the second half of life, beginning around the age of 50 [15], [54], [55]. Studies of idiopathic late onset forms Alzheimer's disease (AD) demonstrate a threefold elevated risk of unprovoked seizures compared with that of the age matched control populations[54]. Patients diagnosed with early-onset forms of Alzheimer's disease appear to be especially vulnerable to seizure, with an 87-fold increase in reported incidence [54]. Several seizure types have been identified in AD, such as temporal lobe spiking, complex partial seizures and nonconvulsive status epilepticus [15], [38], [55]. Both soluble and fibrillar forms of Aβ protein have been implicated in the development of aberrant excitatory network activity. In transgenic mouse models of the disease, over-expression of Aβ protein, which forms hallmark senile plaques associated with AD, results in a hyperexcitable neural network, resulting in silent seizures and epilepsy-like symptoms [36], [37]. Furthermore, a variety of biologic feedback pathways, pre-and post-synaptic, regulating Aβ production have been suggested to contribute to the complex alterations in network activity, accompanied by an activity dependent release of Aβ, that is caused by an excessive activation of glutamatergic NMDA receptor pathways [22], [56]–[60]_ENREF_7_ENREF_7. There remains, however, a critical need to understand how and which forms of Aβ affect synaptic excitation and inhibition, glutamate release, specific ion channel dysfunctions, and circuit level activity.

### Early breakdown of the inhibitory circuits in AD

Traditionally, studies of AD have concentrated on the excitatory cell function but impaired inhibition has also recently been suggested as a potential mechanism of network hyperexcitability [12], [22], [36], [61]. It is conceivable that loss of cortical inhibition and hippocampal hyperactivity of neuronal networks is an early event in AD pathogenesis and is associated with early amyloid deposition in non-demented humans with or without mild cognitive impairment (MCI) [61]. Normal neural function requires finely tuned and balanced excitation and inhibition [62]. For this balance to exist, inhibitory and excitatory neurons must reliably generate action potentials. To generate the action potentials neurons use voltage-gated sodium and potassium channels located in the axon initial segments (AIS) [63], [64]. However, mutations that affect functions of Nav1.1 channel proteins that are expressed in the AIS result in decreased amplitude of action potentials in the parvalbumin-positive subset of inhibitory interneurons [65].

Results obtained in this study suggest that significant hyperexcitability in the perforant pathway activation is due to failure of the inhibitory cells to produce action potentials. This work confirms a recent report of mild interneuron dysfunction associated with early-to-mid-level amyloid pathology, whereby neocortical interneurons had reduced action potential amplitude and this could be rescued with overexpression of Nav1.1 in the inhibitory cells [21]. Alternatively, there have been other channelopathies that have been reported to be disturbed in mouse models of aging and AD, including potassium [66], calcium and calcium-dependent potassium channels [67], [68] and muscarinic M current [69]. Our current results reveal that there is a broad degree of hyperexcitability within the dentate gyrus, the hippocampal circuits and that at the advanced stages of Aβ pathology interneuron ability to generate action potentials may be completely lost. Although we demonstrate that this appears to be the primary mechanism contributing to network hyperexcitability in the APdE9 line, studies in other lines have suggested that selective loss of interneuron populations may be a contributor; however, the APdE9 line have minimal hilar cell loss within the dentate [70]. It is important to note that a small fraction of the interneurons recorded in our studies of aged NTG tissue also showed action potential failures, suggesting that the inhibitory circuit dysfunction may be a part of the normal aging process that is promoted by Aβ deposition. Future studies will need to determine if increased hyperexcitability in the CA1 could be compensated regionally by decreasing excitation or increasing inhibition along the mossy fiber or Schaeffer collateral pathways of the hippocampus proper.

In addition to identifying interneuron failure, we also identified a higher resting membrane potential in the inhibitory interneurons, paralleling to previous reports of higher resting membrane potentials in the excitatory neurons. Decreased resting membrane potentials have been attributed increased membrane conductance in hAPP lines such as APdE9 [16], hypothesized as occurring due to permeabilization of the cell membrane by Aβ. However, in this study we did not observe significant changes in active membrane leakiness in inhibitory neurons as shown by R_in_ quantifications, in spite of a significant amyloid burden in these mice. This suggests that other mechanisms may be of greater importance in regulating polarization of the membrane, such as impaired function of ionic channels [71], buffering of potassium by glia [72], and changes in the extracellular ionic milieu in the advanced plaque deposit stage. Another possibility is impaired ATPase sodium-potassium pump activity due to mitochondrial impairment as has been observed in other models [73].

In summary, these studies demonstrate that interneuron dysfunction occurs normally within a subpopulation of interneurons during the aging process, and this effect appears to be accelerated with the accumulation of Aβ protein. Interneuron dysfunction may be a key pathological event in models of Alzheimer's disease that represents a common mechanism of short-term memory loss; our observations of interneuron dysfunction within the hippocampus of aged APdE9 parallel that observations of cortical interneuron failure recently described in the J20 line by Verret et al [21]. Our work builds upon these initial observations by demonstrating a broad degree of network hyperexcitability within the dentate gyrus of the hippocampus. Combined with interneuron failure, these two events are likely strong contributors to the decline of hippocampal-based spatial learning. It remains to be seen whether the loss of interneuron function in the APdE9 is directly correlated with hyperexcitability due to alterations in channel function, such as seen in the J20 line, or whether this is caused by a different mechanism. Nevertheless, our work suggests that modulation of interneuron activity may be an important therapeutic target for the treatment of early cognitive dysfunctions associated with Alzheimer's disease.
